# Spatiotemporal disturbance characteristics determine functional stability and collapse risk of simulated microbial ecosystems

**DOI:** 10.1038/s41598-018-27785-4

**Published:** 2018-06-22

**Authors:** Sara König, Anja Worrich, Thomas Banitz, Florian Centler, Hauke Harms, Matthias Kästner, Anja Miltner, Lukas Y. Wick, Martin Thullner, Karin Frank

**Affiliations:** 1 UFZ - Helmholtz Centre for Environmental Research, Department of Ecological Modelling, Permoserstraße 15, 04318 Leipzig, Germany; 2 UFZ - Helmholtz Centre for Environmental Research, Department of Environmental Microbiology, Permoserstraße 15, 04318 Leipzig, Germany; 3 UFZ - Helmholtz Centre for Environmental Research, Department of Environmental Biotechnology, Permoserstraße 15, 04318 Leipzig, Germany; 4 Osnabrück University, Institute for Environmental Systems Research, Barbarastraße 12, 49076 Osnabrück, Germany; 5 German Centre for Integrative Biodiversity Research (iDiv) Halle-Jena-Leipzig, Deutscher Platz 5e, 04103 Leipzig, Germany

## Abstract

Terrestrial microbial ecosystems are exposed to many types of disturbances varying in their spatial and temporal characteristics. The ability to cope with these disturbances is crucial for maintaining microbial ecosystem functions, especially if disturbances recur regularly. Thus, understanding microbial ecosystem dynamics under recurrent disturbances and identifying drivers of functional stability and thresholds for functional collapse is important. Using a spatially explicit ecological model of bacterial growth, dispersal, and substrate consumption, we simulated spatially heterogeneous recurrent disturbances and investigated the dynamic response of pollutant biodegradation – exemplarily for an important ecosystem function. We found that thresholds for functional collapse are controlled by the combination of disturbance frequency and spatial configuration (spatiotemporal disturbance regime). For rare disturbances, the occurrence of functional collapse is promoted by low spatial disturbance fragmentation. For frequent disturbances, functional collapse is almost inevitable. Moreover, the relevance of bacterial growth and dispersal for functional stability also depends on the spatiotemporal disturbance regime. Under disturbance regimes with moderate severity, microbial properties can strongly affect functional stability and shift the threshold for functional collapse. Similarly, networks facilitating bacterial dispersal can delay functional collapse. Consequently, measures to enhance or sustain bacterial growth/dispersal are promising strategies to prevent functional collapses under moderate disturbance regimes.

## Introduction

Soil microorganisms provide a variety of important ecosystem services such as litter decomposition, nutrient cycling, climate regulation, and pollutant degradation. However, soil ecosystems are commonly subject to environmental fluctuations that may manifest themselves as recurrent disturbance events. These may limit the ability of microbial communities to remain active and continuously perform essential functions. Disturbances occur with different frequencies as well as with different spatial distribution patterns which can vary down to the pore scale. Only few disturbance types (e.g., temperature changes) affect a site rather homogeneously^1,2^. Most disturbances, however, have a heterogeneous spatial pattern of occurrence, such as flooding or draining events or the release of harmful substances entering the subsurface. Typically, these spatial disturbance patterns are linked to the specific pore network structure and pore size distribution, which can vary substantially among different soils^3,4^. Moreover, pore network structures are not static but can be altered by various causes, for instance, by earth worms and other soil animals, by plant roots and soil fungi, by direct anthropogenic impact such as compaction (e.g., due to vehicles) or mechanical mixing (e.g., during agricultural or forest management activities), or by events such as flooding or freeze-thaw cycles^5–8^. Moreover, several disturbance types (e.g., the contamination with toxic pollutants or antibiotics) depend on the degree of soil water saturation, which itself is often fluctuating^9^. As a consequence, the specific spatial disturbance pattern can vary substantially between recurrent disturbance events. Likewise, the frequency of these disturbances may vary among different ecosystems, with profound consequences. If recovery periods between recurrent disturbance events are too short, the functional performance of a soil microbial ecosystem continuously declines and at some point even collapses, i.e. gets lost permanently^10–12^.

For assessing the potential risk of such functional collapses, the underlying key factors need to be identified. These factors include characteristics of the disturbances such as their frequency, intensity or spatial pattern, but also specific properties of the considered ecosystem. These ‘threshold disturbance characteristics’ likely depend on the specific resistance of the considered ecosystem. For instance, several studies have shown that enhanced dispersal (e.g., due to dispersal networks) can increase ecosystem stability^13,14^. The microbial growth rate is also a factor possibly influencing the stability under disturbances^15,16^. De Vries *et al*.^17^ found that bacterial food webs exposed to drought stress are less resistant albeit more resilient than slower-growing fungal-based food webs. Ecosystems which can buffer the effects of a specific disturbance event thus have likely a lower collapse probability even for highly unfavourable disturbance characteristics.

Although the impact of recurrent disturbances on microbial systems has been determined in several experimental studies^18–21^, it is rather challenging to investigate the long-term effects of spatially heterogeneous disturbances on complex systems in laboratory experiments. Computational models have been established as an alternative for investigation of such effects. Bacterial simulation models not necessarily need to reproduce reality in finest possible detail, but aim to increase mechanistic understanding of ecosystem dynamics by isolating certain processes of complex microbial systems and study them in detail. For instance, we are able to simulate arbitrary many scenarios with different habitat types, spatial environments, or process parameters; while having full control on all system parameters. A higher systems’ understanding then helps to formulate hypothesis for future experimental work.

Microbial simulation modelling already proved valuable, for example, for analysing bacterial mobility in various environments^22–25^, biodegradation efficiency^26^, dynamics during litter decay^27^, microbial dormancy^28^, or bioclogging^29,30^.

Here, we use a numerical simulation approach to investigate the effects of recurrent disturbances varying in their spatial and temporal occurrence on the functional performance and stability of microbial ecosystems (using bacterial pollutant degradation as an exemplary ecosystem function). We explore the key factors determining functional resistance and identify thresholds beyond which functional collapse becomes likely. Particularly, we uncover the interrelated role of (i) disturbance characteristics (frequency and spatial pattern), (ii) microbial properties (specific growth rate and dispersal ability), and (iii) networks facilitating bacterial dispersal (e.g. mycelial networks) for the long-term impact of recurrent disturbances on the biodegradation function.

## Results

**Figure 1:**
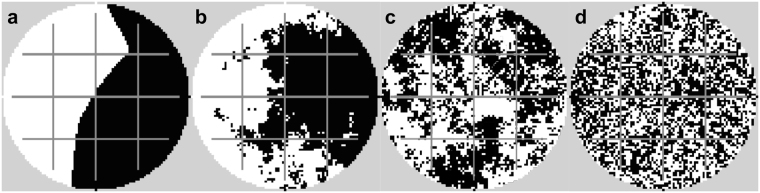
Examples for disturbance patterns with no (**a**, fragmentation parameter *H* = 2), moderate (**b**, *H* = 0.5 and **c**, *H* = 0), and high (**d**, *H* = −1) spatial fragmentation (black: disturbed area, white: undisturbed area). Bacterial dispersal networks (active in selected simulations only) are shown in grey.

For analyzing the stability of the biodegradation performance in response to recurrent disturbance events, we applied a ‘virtual lab’ approach^31^ using the spatially explicit simulation model *eColony*^15,32^. We simulated virtual microbial ecosystems exposed to disturbances introduced as recurring shocks with the effect of a drastic reduction of bacterial biomass in the disturbed area. The spatial disturbance pattern was randomly picked for each single disturbance event but always covered 50% of the two-dimensional area (cf. Methods section). We systematically varied disturbance characteristics (frequency and spatial fragmentation, cf. Fig. 1), microbial properties (bacterial growth rate, bacterial dispersal ability), and the presence or absence of networks enhancing bacterial dispersal (cf. Methods section).

### Relevance of disturbance characteristics

**Figure 2:**
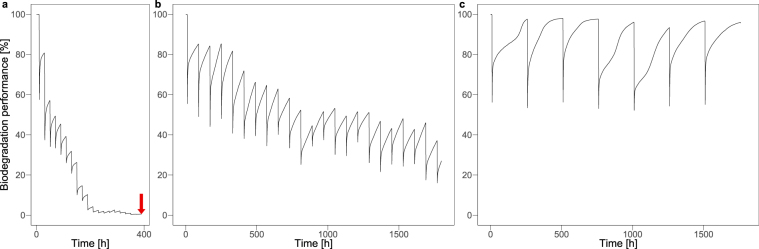
Examples of simulated biodegradation performance over time under recurrent disturbances with moderately fragmented disturbance patterns (*H* = 0.5) and disturbance return interval lengths of 20 (**a**), 80 (**b**), and 250 h (**c**). The arrow indicates functional collapse.

Recurrent disturbances with varying spatial patterns decrease the biodegradation performance of the microbial population depending on the disturbance return interval (i.e. the time between two disturbance events; Fig. 2). For instance, in the case of very frequent disturbances with a return interval length of 20 h (Fig. 2a), this decrease is fast and results in functional collapse after approximately 380 h. In turn, for less frequent disturbances with return interval lengths of 80 and 250 h (Fig. 2b,c), the system is functionally resistant and does not collapse within 2,000 h simulated time. However, the biodegradation performance looks quite different: while the mean performance under disturbances recurring each 80 h decreases substantially compared to the undisturbed reference state, it continuously remains at a relatively high level of performance if disturbances recur only each 250 h.

**Figure 3:**
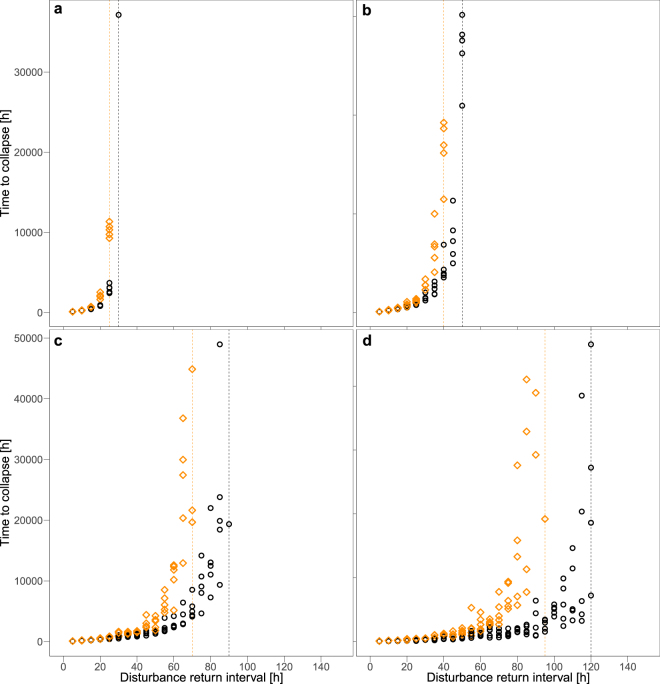
Time to collapse in scenarios with (orange diamonds) and without (black cycles) dispersal networks under disturbance regimes with fragmentation parameter *H* = 2 (**a**), *H* = 0.5 (**b**), *H* = 0 (**c**), and *H* = −1 (**d**) of the disturbance pattern. For each disturbance return interval 5 independent simulation runs were performed. Note that only collapses occurring within the maximum simulated time of 50,000 h are shown. Dashed lines indicate threshold disturbance return interval lengths above which no collapse was observed until the end of the simulated time.

Systematically varying the disturbance return interval length clearly reveals that longer intervals increase the time to collapse as could be expected (Fig. 3). In addition, the time to collapse depends on the degree of spatial fragmentation of the disturbance pattern. Less fragmented disturbance patterns quickly lead to functional collapse even if the disturbance return intervals are long (Fig. 3a,b), whereas higher fragmentation allow the system to withstand higher disturbance frequencies (Fig. 3c,d). For non-fragmented disturbance patterns (*H* = 2), the system only avoids functional collapse if the disturbance return interval is above 120 h. With increasing spatial fragmentation, the threshold disturbance return interval length decreases from 90 h (*H* = 0.5) to 50 (*H* = 0) and 30 h (*H* = −1) for the highest fragmentation. For return interval lengths slightly below or at these thresholds, functional collapse is likely, but the average simulated time until it occurs is often very long (up to 50,000 h, cf. Fig. 3). Only for the highest fragmentation (*H* = −1), there is a distinct separation between either a quick (for interval lengths below the threshold) or almost no functional collapse (for interval lengths at/above the threshold).

**Figure 4:**
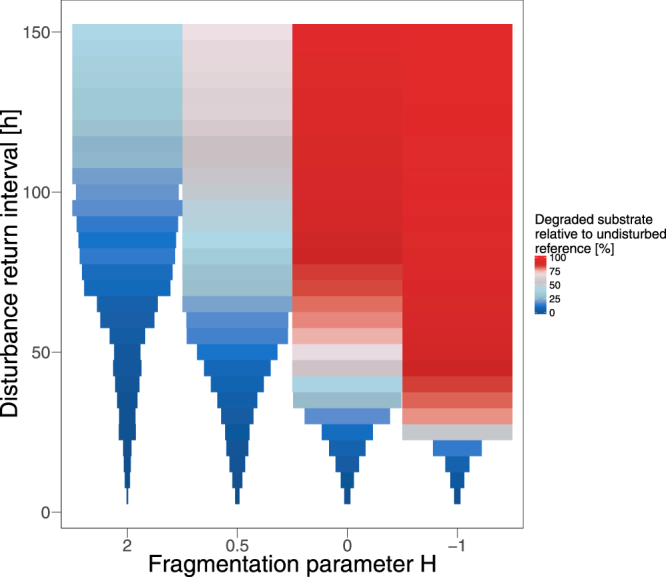
Simulated biodegradation performance during 2,000 h under different disturbance regimes. Box color shows mean biodegradation performance over 10 simulation runs relative to the undisturbed reference scenario. Box width indicates mean time to collapse. If no collapse occurred, boxes have maximal width (equivalent to 2,000 h).

A similar relationship exists between the degree of spatial fragmentation of the disturbance pattern and the functional resistance of biodegradation within a simulated time period of 2,000 h (Fig. 4). With increasing disturbance return interval length, the system’s resistance to recurrent disturbances improves in two aspects: a collapse is less likely and the biodegradation performance is enhanced. Again the degree of spatial fragmentation of the disturbance patterns is important. Decreasing fragmentation (i.e. increasing *H*) reduces the resistance of the biodegradation performance and increases the threshold return interval length for functional collapses. In particular, if the disturbance patterns are highly fragmented (Fig. 4, *H* = −1), only very frequent disturbance events (recurring after max. 20 h) cause functional collapse. These very frequent disturbances reduce the overall biodegradation performance within the simulated 2,000 h to less than 20% of the performance in the undisturbed reference scenario. However, slightly longer disturbance return intervals of 25 h already raise the biodegradation performance to more than 50%. With decreasing fragmentation of the disturbance patterns, the threshold disturbance return interval length, above which collapses do not occur, is increasing up to 100 h for the lowest fragmentation (Fig. 4, *H* = 2). For the latter, even the highest biodegradation performance (observed for the longest disturbance return intervals of 150 h) reaches only approximately 40% of the undisturbed reference.

### Relevance of microbial properties

**Figure 5:**
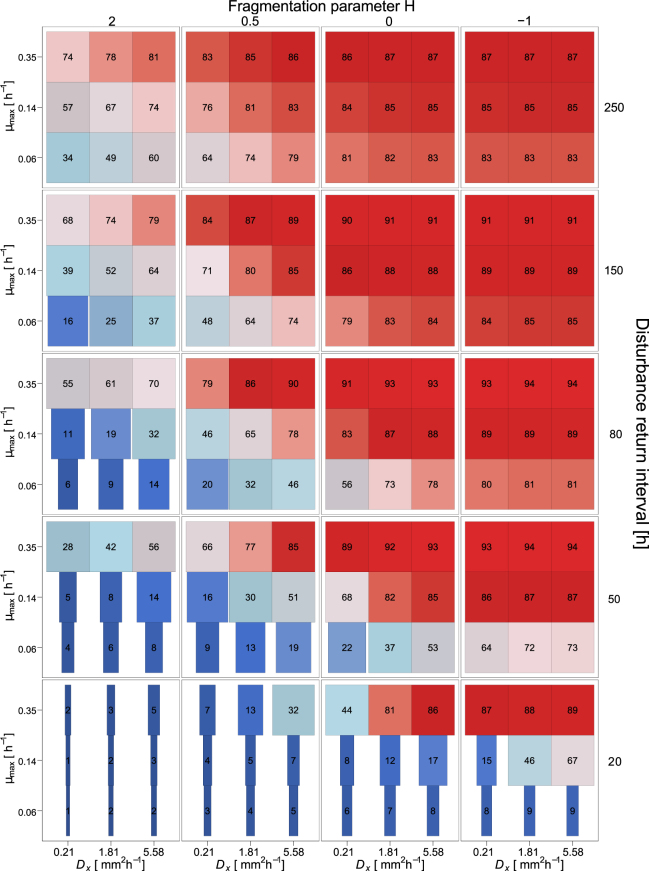
Simulated biodegradation performance during 2,000 h under different disturbance regimes (outer axes) and with different bacterial growth and dispersal behaviour (inner axes). For each disturbance regime, the maximum specific bacterial growth rate *µ*_*max*_ (inner y-axes) and maximum bacterial diffusion coefficient *D*_*x,max*_ (inner x-axes) are varied as described in the Method section. Colors show mean biodegradation performance over 40 simulation runs relative to the undisturbed reference scenario, also given by numbers (in %). Bar widths show mean time to collapse relative to the maximum simulated time of 2,000 h. Maximal width indicates no collapse.

Varying the maximum specific bacterial growth rate *µ*_*max*_ and maximum bacterial diffusion coefficient *D*_*x,max*_ (controlling the dispersal ability of the bacteria) reveals that the relative importance of these two microbial properties - growth rate and dispersal ability - for functional resistance strongly changes with the characteristics of the selected disturbance regimes (Fig. 5). Three zones of different effects on the mean biodegradation dynamics can be distinguished. First, high functional resistance and no risk of collapse, irrespective of the details of the microbial properties, occur in case of disturbances with high to moderate spatial fragmentation and return interval lengths above 80 h (mostly red subplots in Fig. 5). Second, a transition zone occurs within a critical range of disturbance return interval length and spatial fragmentation (subplots with colors varying in x- and y-direction in Fig. 5). Here, functional resistance clearly depends on both microbial properties, and functional collapses emerge under certain but not all disturbance regimes. This range of moderate disturbance regimes shifts to longer return interval lengths with decreasing fragmentation of the disturbance pattern. Third, very low functional resistance always leading to collapse is observed when disturbance return interval lengths are short and the degree of fragmentation is low (dark blue subplot in Fig. 5). The behaviour in this zone is again almost independent of the details of both microbial properties, because the disturbed system cannot resist these very harsh conditions. These influences of the varied microbial properties are also reflected by the corresponding standard deviations (SD) of functional performance (over simulation runs describing the same disturbance scenario but different microbial parameter values, respectively; Fig. S1). Here, the low standard deviations in both the first zone (high functional resistance) and the third zone (low functional resistance), indicate that the microbial parameter values have no considerable influence on biodegradation performance under these disturbance regimes. In the transition zone, however, altered microbial properties can substantially affect biodegradation performance. In these cases of moderate disturbance regimes, the extent of functional recovery within the disturbance return intervals considerably depends on the bacterial ability to disperse into and to regrow in disturbed areas (grey segments in Fig. S1a). In particular, variations in the maximum specific bacterial growth rate *µ*_*max*_ substantially affect the functional resistance to moderate disturbance regimes (SD up to 41%, Fig. S1b; cf. also the enhancement of mean biodegradation performance by up to 80% from the lowest to the highest *µ*_*max*_, bottom right subplot in Fig. 5). The impact of varying the bacterial diffusion coefficient *D*_*x,max*_, on the other hand, is generally lower (maximum SD of 11%, Fig. S1c). Nevertheless, for specific disturbance regimes and *µ*_*max*_ values, also the dispersal ability may strongly influence the mean biodegradation performance (cf. enhancement by 52% from the lowest to the highest *D*_*x,max*_, bottom right subplot in Fig. 5).

### Relevance of dispersal networks

The impact of bacterial dispersal networks on functional performance and collapse also varies with the disturbance regime. Depending on the disturbance return interval length and the degree of spatial fragmentation of the disturbance pattern, dispersal networks may delay or even prevent a functional collapse (Fig. 3). If the disturbance return interval lengths are either very short or very long, dispersal networks do not substantially influence the mean time to collapse. But for intermediate values of the disturbance return interval lengths (value ranges specific for each degree of spatial fragmentation) a significant delay of functional collapse due to dispersal networks can be observed. Moreover, the threshold disturbance return interval lengths above which functional collapse does not occur shift to lower values for all degrees of spatial fragmentation (shifts of orange compared to black vertical lines in Fig. 3a–d, respectively). The highest benefits from dispersal networks are found for non-fragmented disturbance events (Fig. 3a). Here, the functional collapse is on average delayed by 500 h and the time to collapse more than doubled when the disturbance return interval length is 35 h. For longer return intervals, this delay can reach up to 40,000 h and the time to collapse is increased by up to a factor of 16 (e.g. for disturbance return interval of 95 h). If the disturbance return interval length exceeds the threshold of 95 h (i.e. clearly below the threshold of 120 h without networks), the dispersal networks prevent functional collapse.

**Figure 6:**
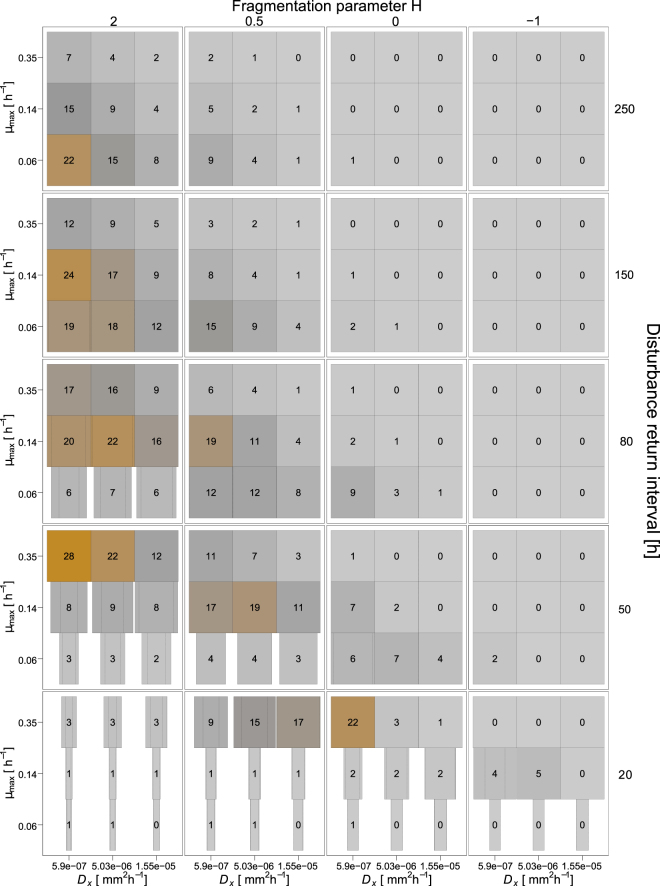
Differences in biodegradation performance during 2,000 h between simulation runs with and without dispersal networks under different disturbance regimes (outer axes) and with different bacterial growth and dispersal behaviour (inner axes). For each disturbance regime the maximum specific bacterial growth rate *µ*_*max*_ (inner y-axes) and maximum bacterial diffusion coefficient *D*_*x,max*_ (inner x-axes) are varied. Colors show improvement of mean biodegradation performance over 40 simulation runs relative to the undisturbed reference scenario, also given by numbers (in %). Box widths show mean time to collapse with dispersal networks, thin dashed box widths show mean time to collapse without dispersal networks, both relative to the maximum simulated time of 2,000 h. Maximal width indicates no collapse.

Furthermore, dispersal networks increase the mean biodegradation performance mainly in cases belonging to the transition zone of moderate disturbance regimes (i.e. where the microbial properties have an influence on the functional resistance, cf. above) and if the maximum bacterial diffusion coefficient is low (Fig. 6). Here, the significantly enhanced bacterial dispersal along the networks increases the mean biodegradation performance by a factor of 2 (from 28 to 56% of the undisturbed reference performance for the disturbance return interval length 50 h, *H* = 2, *µ*_*max*_ = 0.35 h^−1^, *D*_*x,max*_ = 0.21 mm^2^ h^−1^, Fig. 6).

Mostly, the disturbance regimes under which dispersal networks can increase functional resistance are those that also show effects when varying the parameter value *D*_*x,max*_ which determines bacterial dispersal ability without networks (cf. Figs 5, S1c). However, for some cases of fragmented disturbance patterns (low values of *H*), an increased bacterial diffusion coefficient (0.21 to 5.58 mm^2^ h^−1^) improves biodegradation performance, while further increasing the bacterial dispersal ability along the networks does not have this effect (cf. Figs 5, 6 for *H* = −1). Altogether, bacterial dispersal networks can strongly enhance the functional resistance to recurrent disturbances but only for disturbance regimes within a specific range of spatiotemporal characteristics.

## Discussion

With our simulations of recurrent disturbance events affecting a simplified microbial ecosystem, we identified the disturbance characteristics as key factors influencing the long-term functional stability in terms of the biodegradation performance and the probability of a functional collapse. These characteristics include the disturbance return interval length and the spatial fragmentation of disturbance patterns. In addition microbial properties in the given ecosystem (bacterial growth rate, and bacterial dispersal ability) and the presence of bacterial dispersal networks (representing the effect of mycelial networks) may also influence functional stability under specific circumstances. By systematically varying all these factors, we showed that they are often highly interrelated and were able to disentangle their effects.

If the return interval length of spatially varying disturbance events is shorter than a critical threshold, the biodegradation performance declines until it eventually collapses. This threshold, in turn, depends on the degree of spatial fragmentation of the disturbance pattern. If the disturbance patterns are highly fragmented, even rather short disturbance return intervals still allow for long-term functional resistance. Here, the mean distance between disturbed and undisturbed habitats is low and, thus, the disturbed habitats are recolonized quickly. This allows for high functional recovery in the disturbed area between individual disturbance events, such that no functional collapse occurs and the overall biodegradation performance remains high. In a previous study, we showed that the functional recovery after a single disturbance event is significantly faster if the disturbance area is highly fragmented^15^. Here, we showed that this faster recovery after highly fragmented disturbances facilitates a higher resistance against recurrent disturbances of this spatial characteristic, even if the disturbances recur very frequently (e.g. H = −1, and disturbance return interval higher than 30 h, cf. Fig. 4). This is in agreement with experimental studies showing that recolonization from disturbed to undisturbed habitats can lead to a higher resistance under recurrent disturbances^18,33^. If the disturbances occur in less fragmented disturbance patterns, the ecosystem function collapses already for moderate disturbance frequencies. This is due to the increasing mean distance between disturbed and undisturbed habitats. Here, the bacteria need more time for recolonizing all of the disturbed habitats. In consequence, also the overall biodegradation performance is low in these cases because the effects of the individual disturbance events accumulate. If, in these cases, also the disturbance return interval length is short, more and more bacterial habitats are substantially affected, their contribution to biodegradation declines, and the function cannot be maintained leading to collapse. This result is in line with an experimental study assessing how drying-rewetting cycle frequencies influence microbial activity, in which cumulative effects of recurrent disturbances resulted in a functional collapse^12^. Comparable to our findings, more frequent disturbance events increased the probability of functional collapse. In another experimental study, Garnier *et al*.^34^ showed that the recovery time of an aquatic microbial community increases with increasing number of disturbances, indicating a cumulative effect as well. Similar to this, Jurburg *et al*.^35^ observed multiplicative effects of different temperature shocks on the recovery of soil microbial communities.

Our simulation results are also directly linked to an ongoing debate in general ecology on the relevance of explicit spatial occurrence patterns of disturbances for population extinction risks^36–39^. For instance, the extinction probability of locally dispersing populations under recurrent disturbances increased with spatial correlation of disturbances in a theoretical modelling study^38^. Kallimanis *et al*.^36^ proved that increasing spatial aggregation of disturbances increases the risk of mass extinction in a spatially explicit metapopulation model. Our general conclusions on the relevance of spatial fragmentation of disturbances for resistance are in line with these findings. However, in particular the role of spatially correlated disturbances as driving force for functional collapse has received much less attention.

Enhanced properties of the microorganisms in terms of bacterial growth and dispersal can buffer the accumulated effects of the recurrent disturbances to a certain extent. This applies, however, only under moderate disturbance regimes denoted by a certain trade-off range between short return intervals but high spatial fragmentation or longer return intervals but lower spatial fragmentation (Fig. 5). Outside this range, the disturbance regime is either too severe, such that the functional resistance of the system is low regardless of the specific properties of the bacteria. Or the disturbance regime is weak, such that the disturbance events are buffered quite well and the function is maintained in all tested scenarios, irrespective of how fast the bacteria grow or disperse. Although we tested the growth rate in a much smaller range than bacterial dispersal (varying by a factor of up to 6, compared to a factor of up to 22 in case of the bacterial diffusion coefficient), the rate of bacterial growth proved to be more important for the functional resistance than the velocity of bacterial dispersal. A higher growth rate allows for a faster local recovery and, in consequence, functional stabilization of the disturbed habitats. Here, the overall biodegradation performance is enhanced, and functional collapse occurs less often. Our finding that high bacterial growth rates can increase the functional stability, dependent on the specific disturbance regime, is supported by observations made in other modelling as well as experiment-based studies^40–42^. By using a spatial population dynamics model, Fuller *et al*.^41^ examined the cumulative impact of climate and harvest, and proved that populations’ persistence is mostly driven by growth. Frenk *et al*.^40^ highlighted the importance of higher growth rates for the functional stability by experimentally testing the response of soil bacterial communities to disturbances in presence of different types of irrigation water. They showed that functional recovery after a heat disturbance was increased under nutrient-rich conditions favoring species with higher growth rates. In a microcosm experiment with two connected ecosystems consisting of a heterotroph and an autotroph community, Harvey *et al*.^42^ observed collapses in terms of inhibited detritus flow if species growth rates are slow and the disturbance regime is severe.

It is known from general ecology, that enhanced dispersal can increase ecosystems’ resistance^43–47^. In a previous study, we identified bacterial dispersal as key for functional long-term resistance under spatially heterogeneous recurrent disturbances, if these always occur with the same spatial disturbance pattern^48^. In this case, the continuously undisturbed area serves as a pool of bacteria, which can disperse into the disturbed areas and help buffering the following disturbances. Novel in our study is that each disturbance is applied with a different spatial disturbance pattern. Thus, no area remains completely undisturbed and bacterial dispersal gets less important than bacterial growth, as no bacteria are left to disperse from an undisturbed pool, and they thus have to regrow for recovering. If they are able to regrow (e.g., due to a high growth rate), enhanced bacterial dispersal helps to further increase the functional stability.

In previous experimental work, we showed that bacterial dispersal networks (mimicking facilitated bacterial dispersal along mycelia) increase the functional resistance of biodegradation when exposed to osmotic stress^14^. Our present simulation results indicate that dispersal networks also enhance the long-term resistance under recurrent disturbances. These networks reduce the threshold time spans between single disturbance events required to prevent functional collapse. This means that with dispersal networks the biodegradation function is maintained under certain disturbance frequencies for which it would otherwise collapses. This benefit occurs for all degrees of spatial fragmentation of the disturbance patterns and is most pronounced under non-fragmented disturbances. Under those disturbance regimes, the long mean distances between disturbed and undisturbed habitats prolong the recovery time from single disturbance events. By helping the bacteria to overcome long distances and quickly reach the interior of recently disturbed areas, the dispersal networks enable functional resistance to more frequent disturbances. Furthermore, under certain disturbance regimes, functional collapses are considerably delayed, although not completely prevented, by these effects of bacterial dispersal networks. However, the overall biodegradation performance is substantially increased in presence of bacterial dispersal networks under certain conditions. Again, these improvements depend on the disturbance regime. Under certain moderately intense disturbance regimes, the biodegradation performance is increased by dispersal networks, especially if the spatial fragmentation of the disturbance pattern is low and bacterial dispersal slow. In some cases, bacterial dispersal networks cause a shift of the threshold for functional collapse, but do not substantially enhance the biodegradation performance. Here, the time to collapse indicates an increased functional resistance due to dispersal networks, whereas the assessed biodegradation performance indicates no benefit. This is because the biodegradation performance is maintained without collapsing, but on a very low level. In consequence, the time to collapse alone is not an indicator for the functional resistance under recurrent disturbances, and, it may be appropriate to define some minimum biodegradation performance level. We may then refer to a system as ‘functionally resistant’ if this minimum level is constantly exceeded under recurrent disturbances.

Our simulation analyses suggest that it is crucial to consider the following aspects to assess the stability of contaminated soils under recurrent disturbances: the characteristics of present disturbance regimes, especially its spatial pattern, and certain microbial properties in the given ecosystem, such as bacterial growth rate and dispersal ability. Since these different factors are strongly interrelated, future experimental studies should assess the influence of the disturbance characteristics on functional resilience including microorganisms with different properties with a focus on spatial effects. We hypothesise that recurrent disturbances with a moderate frequency or spatial correlation have less severe effects if species have higher growth rates. Very frequent or non-fragmented disturbances, however, cannot be compensated by advantageous species properties. Here, also soil-based experiments testing the effects of mechanical mixing (i.e. increasing the soil pore network’s spatial fragmentation) on microbial ecosystem dynamics may be important for determining key factors for functional stability.

We found a high importance of the bacterial growth rate, but only for specific disturbance regimes. Hence, established strategies for enhancing bacterial growth, such as additional supply of nutrients, increase of oxygen concentration in the soil water or stimulation of plant root growth (which enhances bacterial growth by producing exudates acting as a carbon source for the bacteria)^49–51^, may not improve the biodegradation performance under very frequent or rare disturbance events. On the other hand, the functional benefits caused by improving bacterial dispersal (e.g., by increasing the soil water content or stimulating dispersal networks) may also depend on the specific disturbance and not always be effective.

Using our spatially explicit model, a ‘virtual lab’ approach allowed us to simulate various disturbance regimes characterized by different spatial occurrences and frequencies. Indeed, spatial heterogeneous disturbance patterns occurring in constant return intervals combined with arbitrarily varying microbial properties can hardly been achieved in laboratory experiments. For identifying thresholds for functional collapse, a very long simulation time was necessary. This would be inefficient in terms of time and resources in experimental work, but gives hints for the relevant time-scale when performing such collapse experiments. We were also able to define a range of disturbance characteristics, under which microbial properties are essential factors for stabilizing a function. However, further experimental work is needed to confirm our findings and increase the understanding of the functional response to recurrent disturbances in complex natural ecosystems.

## Methods

### Simulation model eColony

#### Model description

We applied a spatiotemporally explicit, ecological model (named *eColony*) developed in a recent study^15^. The model describes bacterial growth, bacterial dispersal and substrate consumption (representing biodegradation of organic compounds) with a set of reaction-diffusion equations. It is an extension of an established model which was parametrized based on laboratory experiments^32,52^. The model operates on a two-dimensional circular domain with diameter of 88 mm and reflective boundaries, consisting of rectangular habitats of 1 mm² size each. The model equations are solved using a finite difference method^32,53^ and time steps of 1 minute, implemented in the programming language Delphi.

Each simulation step includes the following processes: substrate uptake by bacteria, uptake allocation to energy-requiring tasks, bacterial dispersal, growth and reproduction, substrate diffusion, and substrate input. Substrate input was implemented by assuming an area-wide permanent input allowing for the substrate to accumulate up to a maximum substrate concentration *C*_*s,max*_, representing subsequent dissolution of an adsorbed compound, cf.^54,55^. Substrate is then refilled in each time step depending on the difference between maximum substrate concentration *C*_*s,max*_ and current substrate concentration *C*_*s*_ multiplied by the substrate input rate parameter λ (cf. Eq. 2).

For simulating dispersal networks acting as highways for the bacteria, corridors of habitats with a higher diffusivity are implemented (cf. Fig. 1). If this model feature is activated, the bacteria can disperse much faster in the habitats belonging to the network.

#### Equations

The dynamics of bacterial colony growth and substrate degradation are described using the following reaction-diffusion equations:

1 $$\frac{\partial Cx}{\partial t}=\nabla ({D}_{x}({{C}}_{x}{,}{{C}}_{s})\nabla {{C}}_{x})+({q}({{C}}_{s}){YG}-{a}-{d}({C}x,{{C}}_{s})){{C}}_{x,}$$

2 $$\frac{\partial {C}_{s}}{\partial t}={D}_{s}{\nabla }^{2}{C}_{s}+{\rm{\lambda }}({C}_{{s},{\max }}-{{C}}_{s})-{q}({{C}}_{s}{)C}_{x,}$$

where *C*_*x*_ and *C*_*s*_ are the concentrations of bacteria (g_x_ mm^−2^) and substrate (g_s_ mm^−2^), *D*_*x*_ and *D*_*s*_ are the diffusion coefficients of bacteria and substrate (mm^2^ h^−1^), *q* is the specific substrate uptake rate of bacteria (g_s_ g_x_^−1^ h^−1^), calculated according to $q{=}{{q}}_{{\max }}\frac{{{C}}_{{S}}}{{{C}}_{{S}}{+}{{K}}_{{S}}}$ with *q*_*max*_ as the maximum specific uptake rate (g_s_ g_x_^−1^ h^−1^) and *K*_*s*_ is the half-saturation constant (g_s_ mm^−2^). *Y*_*G*_ is the growth yield coefficient (g_x_ g_s_^−1^), *a* is the specific maintenance rate (h^−1^), *d* is the specific dispersal cost, expressed as biomass decrease (h^−1^), and λ is the substrate input rate parameter (h^−1^). Note that both *D*_*x*_ and *d* vary depending on substrate availability, cf.^32,52^.

#### Model validation and assumptions

The model was validated with selected laboratory experiments with *Pseudomonas putida* PpG7 (NAH7) as model organism and glucose as model substrate^52^. Bacterial colonies were grown in Petri dishes on minimal medium agar with an agar concentration of 3, 4, and 5 mg l^−1^ and homogeneously distributed substrate (0.1 and 1 g l^−1^ glucose) in four replicates, respectively. After inoculation, the growing colonies were observed for 66 h by hourly image scanning using a commercial flatbed scanner (HP Jetset 7400c). The total area of the bacterial colonies was then calculated with the image analysis software ImageJ. According to the experimentally derived results, the maximum bacterial diffusion coefficient *D*_*x,max*_ for each agar concentration were fitted by direct search optimization. Diffusion coefficients on dispersal networks were fitted according to laboratory experiments using disposable polymer coated glass fibres for simulating the properties of fungal hyphae to build a continuous water film^32^.

Based on these laboratory experiments and other established modelling approaches from literature, we made several assumptions for our model. The effective growth rate depends on the present substrate concentration which controls the effective uptake rate via Monod kinetics^56,57^ (cf. Eq. 1), with a higher substrate concentration resulting in a higher effective growth rate. Active bacterial mobility is implemented depending on the local substrate concentration as well^52^. Dispersal is inhibited, if the substrate supply by the extant substrate concentration is lower than the fixed maintenance rate *a*. Otherwise, bacterial dispersal increases with increasing substrate concentration up to the maximum diffusion coefficient *D*_*x,max*_. Those conditional dispersal strategies are widely discussed in general ecology and successfully applied in several modelling approaches^58–60^.

### Disturbance scenarios

As undisturbed reference state, we calculated a spatially homogeneous steady state in which the maintenance demand of the bacterial population exactly matches the substrate input (Table 1). Consequently, the steady state bacterial population receives enough energy for survival but not for growth and, therefore, remains constant. These steady state concentrations were used as initial condition of bacterial biomass and substrate concentration.

**Table 1:** Base set of model parameter and initial conditions.

Parameter/State variable	Symbol	Value	Unit^a^	Source
Maximum specific growth rate	*µ*_*max*_	0.1386	h^−1^	^14^
Specific maintenance rate	*a*	0.0003	h^−1^	^32^
Growth yield	*Y*_*G*_	0.6	g_x_ g_s_^−1^	^32^
Maximum substrate uptake rate	*q*_*max*_	0.2315	g_s_ g_x_^−1^h^−1^	${{\rm{q}}}_{{\rm{\max }}}=\frac{{{\rm{\mu }}}_{{\rm{\max }}}+{\rm{a}}}{{{\rm{Y}}}_{{\rm{G}}}}$
Half-saturation constant	*K*_*s*_	4.439E-07	g_s_ mm^−2^	^32^
Maximum bacterial diffusion coefficient	*D*_*x,max*_	0.212	mm^2^ h^−1^	^52^
Maximum bacterial diffusion coefficient along dispersal networks	*D* ^*dn*^_*x,max*_	144	mm^2^ h^−1^	^32^
Substrate diffusion coefficient	*D*_*s*_	2.326	mm^2^ h^−1^	^62^
Substrate input rate	*λ*	0.24	h^−1^	^54^
Initial bacterial concentration	${C}_{x}^{\ast }$	2.366E-4	g_x_ mm^−2^	undisturbed reference state
Initial substrate concentration	${C}_{s}^{\ast }$	3.847E-11	g_s_ mm^−2^	undisturbed reference state

^a^g_x_ – grams of dry biomass, g_s_ – grams of substrate,

Pulse disturbance events were introduced as shock events reducing the bacterial biomass concentrations within a defined disturbance area by multiplying with a factor of 10^−9^ (i.e. surviving biomass of 2.366e^−13^ g mm^−2^ which is roughly equivalent to 1 surviving bacterial cell in each habitat after the first disturbance). Bacteria in the undisturbed area were not directly affected by disturbance events. We did not consider any microbial adaptation processes in response to the disturbance events.

We used the midpoint displacement algorithm for generating the spatial distribution patterns of the disturbance area with the fragmentation parameter *H* and proportion size *p*, see eg.^61^. For all simulations, the size of the disturbance area (i.e. the proportion size *p*) was set to 50% of the simulation domain. The fragmentation parameter *H* was set to −1, 0, 0.5, or 2 for representing the full range between the extreme situations of ‘highly fragmented’ (*H* = −1) and ‘non-fragmented’ (*H* = 2) disturbance patterns. Example patterns with the selected degrees of fragmentation are shown in Fig. 1.

Disturbance events were repeatedly applied after a defined disturbance return interval, which was kept constant within each simulation run (i.e. disturbance events recurred at a constant frequency). The length of the disturbance return interval was varied between different simulation scenarios covering a range from 5 to 250 h. Within each simulation run, disturbance events were applied with the same degree of fragmentation but each event with an individual explicit spatial disturbance pattern for simulating stochastic disturbance events. The results for each disturbance regime (i.e. a specific combination of spatial fragmentation parameter *H* and return interval length) are presented as averages over 5, 10, or 40 independent simulation runs (see respective figure captions), each with a different set of random realizations of spatial disturbance patterns.

In a first set of scenarios, simulations were performed considering a base set of parameter values determining bacterial growth and dispersal (Table 1). Presented results were derived with these parameter values if not mentioned otherwise. Subsequently, we performed simulations where we varied certain parameter values to determine the influence of bacterial growth rate and bacterial dispersal ability on functional performance under the given disturbance regimes. The maximum specific growth rate µ_max_ covered the values 0.347 h^−1^, 0.1386 h^−1^ (base set value), and 0.0639 h^−1^ which correspond to generation times of 2, 5, or 10 h, respectively. The maximum bacterial diffusion coefficient *D*_*x,max*_ was increased from 0.212 mm^2^ h^−1^ (base set value, representing unfavourable bacterial dispersal conditions) to 1.81 mm^2^h^−1^ (intermediate dispersal conditions), and 5.58 mm^2^h^−1^ (favourable dispersal conditions, e.g. due to higher water availability)^52^. Furthermore, each parameter value combination was simulated in absence and in presence of bacterial dispersal networks.

### Analysis

For determining the time to collapse (Fig. 3), simulation runs were either performed up to a maximum simulated time span of 50,000 h or stopped when the overall biodegradation performance permanently diminished, the latter indicating functional collapse. In this case, the respective time point was recorded as the time to collapse. Additionally, we performed shorter simulations (simulated time span of 2,000 h) to determine the functional resistance to disturbances in various scenarios (Figs 4, 5, 6). For this purpose, the total degraded substrate within a 2,000 h time span was used as a measure of the functional resistance to the respective disturbance regime. Standard deviations of the total degraded substrate within subsequent disturbance regimes (i.e. same disturbance characteristics of return interval and degree of fragmentation) were calculated for analyzing the influence of the microbial properties (bacterial growth rate and bacterial dispersal ability) and the dispersal networks on functional resistance. All plots were produced with the package ggplot2 of the statistical computing language R, version 3.3.1.

### Data availability

The datasets generated and analysed during the current study are available from the corresponding author on request

## Electronic supplementary material

Supplementary Information
