# Supplementary material for: Spatiotemporal disturbance characteristics determine functional stability and collapse risk of simulated microbial ecosystems
Source: Sci Rep. 2018 Jun 22;8:9488. doi: 10.1038/s41598-018-27785-4 (PMC6015006; doi:10.1038/s41598-018-27785-4)
Supplement: Supplementary file 1 — Supplementary Information [file 41598_2018_27785_MOESM1_ESM.pdf]

<u>Supplementary Information</u>:

Spatiotemporal disturbance characteristics determine functional stability and collapse risk of simulated microbial ecosystems

Sara König[1,2,4]*, Anja Worrich[2,3], Thomas Banitz[1], Florian Centler[2], Hauke Harms[2,5], Matthias Kästner[3], Anja Miltner[3], Lukas Y Wick[2], Martin Thullner[2] and Karin Frank[1,4,5]

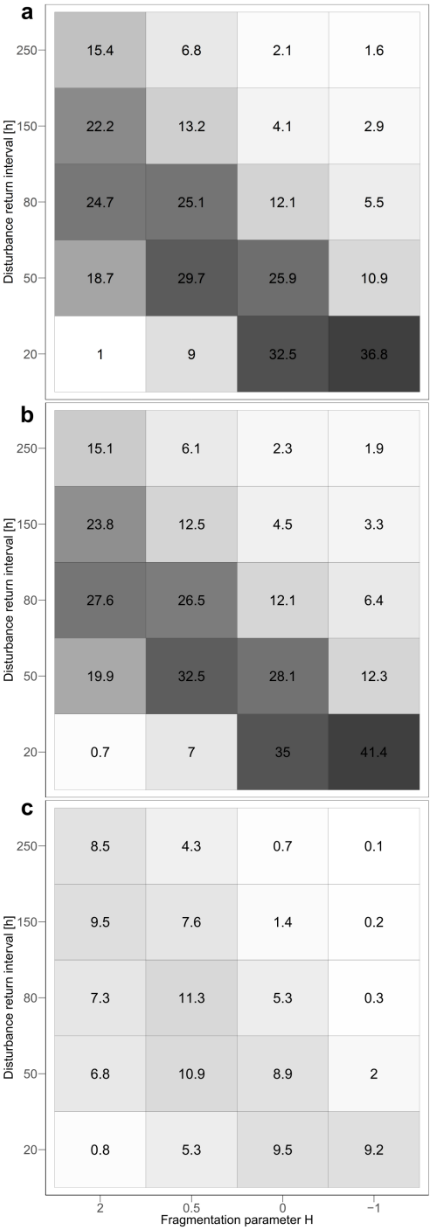

Supplementary Figure S1: Standard deviation (SD) of simulated biodegradation performance over 2,000 h with different bacterial growth and dispersal behavior, for each disturbance regime shown in Fig. 4. For each disturbance regime, (a) SD over all 360 simulation runs (i.e. 40 simulation runs per combination of maximum specific bacterial growth rate μ_{max} and maximum bacterial diffusion coefficient $D_{x,max}$, cf. Fig. 4), (b) SD over 120 simulation runs for each value of $D_{x,max}$, respectively (i.e. only effects of varying μ_{max} with 40 simulation runs per μ_{max} value), and (c) SD over 120 simulation runs for each value of μ_{max}, respectively (i.e. only effects of varying $D_{x,max}$, 40 simulation runs per $D_{x,max}$ value) are shown.
